# Overgrowth Syndromes—Evaluation, Diagnosis, and Management

**DOI:** 10.3389/fped.2020.574857

**Published:** 2020-10-30

**Authors:** Joshua Manor, Seema R. Lalani

**Affiliations:** Department of Molecular Genetics, Baylor College of Medicine, Houston, TX, United States

**Keywords:** overgrowth, Beckwith-Wiedemann, Simpson-Golabi-Behmel, Sotos, Weaver, Pten, PIK3CA, Proteus Syndrome

## Abstract

Abnormally excessive growth results from perturbation of a complex interplay of genetic, epigenetic, and hormonal factors that orchestrate human growth. Overgrowth syndromes generally present with inherent health concerns and, in some instances, an increased risk of tumor predisposition that necessitate prompt diagnosis and appropriate referral. In this review, we introduce some of the more common overgrowth syndromes, along with their molecular mechanisms, diagnostics, and medical complications for improved recognition and management of patients affected with these disorders.

## Introduction

Healthy growth can be defined as a progression of changes in height, weight, and head circumference and is predicted to follow standardized *growth curves*, reflecting the overall health and nutritional status of an individual (1). As early as the 18th century, the importance of growth charts was recognized, and its role as a diagnostic tool is now widely appreciated (2). Nowadays, growth is routinely followed prenatally with serial ultrasonography (defined as changes between consecutive measurements of biparietal diameter, abdominal circumference, and femur length). Following conception, the fetus follows a rapid growth phase around 13–16 weeks of gestation which gradually slows down until birth (3). Postnatally, linear growth velocity declines to 15–17 cm/year in the first 2 years of life, which further decreases until puberty to about 5 cm/year. At puberty, linear growth velocity peaks at 6–10 and 5–11 cm/year for girls and boys, respectively (4). This accounts for a final height of 153–174 cm (60–68.5 inches) for girls and 165–189 cm (65–74 inches) for boys falling between then 5th and 95th centiles, according to the CDC standardized growth charts (5). The final height, which is the result of a complex interplay among transcription factors, hormones, and a large variety of target cells that lasts for about 18 years, eventually falls within a 19–24 cm (7.5–9.4 inches) range for the vast majority of the population. This accounts for only 12–14% variability in final height, compared with a 56–66% variability in final weight.

Skeletal growth occurs in the epiphyseal plate of long bones owning to the unique differentiation state of chondrocytes (6, 7): resting chondrocytes differentiate into proliferating chondrocytes, which in turn differentiate further into hypertrophic chondrocytes. Proliferating chondrocytes secrete extracellular matrix (ECM) components to support the bone structure, while hypertrophic chondrocytes apoptose and promote osteoblast influx responsible for bone mineralization. The differentiation process is regulated both by paracrinic and endocrinic hormonal axes. Proliferation of chondrocytes in the growth plate is upregulated by Indian hedgehog (IHH), which stimulates PTH-related protein (PTHrP), and specific bone morphogenic protein (BMP) and is repressed by the fibroblast growth factor (FGF)—FGFR3 receptor pathway (overactivation of FGFR3 results in achondroplasia). Chondrocyte hypertrophy is stimulated by thyroid hormones via the Wingless-int 4 (Wnt 4) β-catenin pathway; it is inhibited by the IHH–PTHrP pathway. Both hypertrophy and proliferation are stimulated by the growth hormone (GH)–insulin-like growth factor 1 (IGF-1) pathway (8). Estrogen, when secreted in high dose in puberty, promotes growth plate closure by depletion of proliferation and promoting hypertrophic chondrocytes' death (9). Excess of glucocorticoids suppresses IGF-1 proliferative signal (10) and proinflammatory cytokines induce chondrocyte apoptosis and suppress skeletal growth (11).

For abnormally short stature (with or without poor weight gain), many publications aimed at guiding practitioners are available to assist in a rapid diagnosis [e.g., Bithoney et al. (12), Rose et al. (13), and Jaffe (14)]. Management can be facilitated by the publications of the Pediatric Endocrine Society, which detail the guidelines for initiation of growth hormone therapy (15). The other end of the growth spectrum, tall stature, may be perceived a sign of “healthy growth,” undermining a discussion about possible pathologic processes.

There is no specific definition for tall stature; as commonly used in other specialties, the standard characterization of tall stature is a stature that exceeds 2 standard deviations (SD) above the median growth for the reference population. Individuals who exceed the 95-centile, therefore, are considered to be of tall stature and are much less likely to be brought to medical attention or be evaluated by the relevant subspecialty compared to those with short stature.

The term *overgrowth* generalizes abnormally tall stature and is used to describe three phenotypes:

- *Prenatal overgrowth*—A phenotype which includes newborns who are large for gestational age (LGA), either macrosomic newborns (>4,000 g), or with length and weight ≥97th centile (16). Common considerations include maternal diabetes and overgrowth syndromes such as Beckwith–Wiedemann syndrome (BWS). Affected individuals may continue to show an accelerated growth postnatally (pre- and post-natal overgrowth) or may grow at a normal pace with length falling within 2 SDs of the mean.
- *Post-natal overgrowth*—This phenotype includes individuals who are noticed to have an accelerated growth pattern starting typically in childhood or adolescence. Childhood onset of excessive growth is usually a manifestation of endogenous hormone-dependent growth, and therefore, this group is consistent with mainly endocrine abnormalities (such as thyroid, growth hormone, sex hormones, or glucocorticoid). Other etiologies include familial tall stature (constitutional tall stature), precocious puberty, obesity, Marfan syndrome, homocystinuria, Klinefelter syndrome, and 47,XYY syndrome (4).
- *Segmental overgrowth*—A phenotype of excessive growth that is confined to one or a few regions of the body, e.g., a single digit, a whole extremity, one side of the face, or the entire head (macrocephaly). It can be expressed as asymmetrical growth of musculoskeletal, adipose, and/or brain tissue along with focal hyperplasia of capillary venous or lymphatic vessels and overlying skin lesions. Patients are more likely to present to medical attention due to the unaesthetic nature of the asymmetric growth. The segmental or mosaic overgrowth often occurs with overactivation mutations of the PI3K/AKT/mTOR (phosphoinositide-3-kinase/protein kinase B/mammalian target of rapamycin) pathway (17). Individuals can be mosaic for these mutations meaning that a genetic change occurred after the formation of the zygote, and only a subset of cells express these mutations. This pathway, often found to be activated in malignancy, is a major growth pathway that responds to several growth factors (GF) like epidermal (EGF), vascular (VEGF), platelet-derived (PDGF), or insulin-like (IGF-1). It is closely related to the Ras/MAPK pathway (17). Deactivating mutation in repressors of the PI3K/AKT/mTOR pathway, for example, phosphatase, and tensin homolog (PTEN) or tuberous sclerosis complex 1 or 2 (TSC1 or 2), can also lead to segmental overgrowth. Macrocephaly, a common finding in segmental overgrowth, requires special attention. Head circumference (HC) ≥ 98%, even in the absence of other findings, may be associated with autism or intellectual disability (18, 19). Specific attention should be given to an increased HC > 3 SD above mean (≥99.7%-ile), which is highly suspicious of PTEN Hamartoma Tumor syndrome, as discussed below.

Overgrowth syndromes can be associated with hormone imbalance, life-threatening hypoglycemia (e.g., BWS), seizures (Sotos syndrome), developmental delay (Sotos syndrome, Weaver syndrome), and an increased susceptibility to malignancy (Wilms tumor, hepatoblastoma, etc.). Clinicians should therefore maintain a high index of suspicion for a prompt diagnosis.

## An Outline For the Approach to a Patient With Suspected Overgrowth

Clinical diagnosis of overgrowth syndrome should be made either with or without parental concerns. There is no established algorithm for evaluation of overgrowth; clinicians should rely on detailed history and physical examination to generate an appropriate differential diagnosis. A key point in the evaluation is the assessment of growth velocity. Auxiliary test can include full blood counts and complete biochemical analysis, IGF-I, IGFBP-3, free T4, and TSH as well as a karyotype study and bone age (20). Bone age, an alias for the individual's biological age, include radiographic image of the left hand and wrist. The radiograph is either compared to an atlas of reference (Greulich and Pyle atlas from 1959), or a bone age is assigned by summation of maturity scores for each individual bone (Tanner-Whitehouse, developed in 1975). Recently, the automated BoneXpert technology was approved in Europe. Abnormal results are a mismatch of 2 standard deviations of the bone age from the chronological age. However, two caveats exist: (i) the inter-observer variance is estimated to be 0.4–0.8 years and (ii) for accurate bone age before the age of 1, hemiskeleton imaging is needed (21, 22). Results therefore must be interpreted in the appropriate clinical context, especially for infants.

Several of the genetic overgrowth syndromes are inherited in an autosomal dominant manner and therefore can “run in the family.” If a clinician suspects a genetic overgrowth syndrome, questioning about family history is of high importance. Clinicians should remember that in case of one affected individual with an autosomal dominant disorder, testing for the familial mutation should be offered to the other affected family members. It also entails 50% chance of transmission to the next generation—it may be considered by some clinicians too early to discuss family planning with a pediatric patient; however, postponing a discussion may cause this information to be lost.

If a genetic syndrome is suspected, genetic testing should be offered to confirm a diagnosis. Genetic testing can be done via blood or saliva test and usually takes 2–6 weeks to result, depending on the type of test. Saliva samples may sound attractive to families, as they do not involve a needle stick; however, for young patients, collecting adequate saliva may be a tedious task. For blood sampling, no fasting is required, and samples can be processed with as little as 2 ml of blood. Analysis of suspected genes usually includes sequencing of the gene(s) of interest plus deletion and duplication analysis of the gene(s), also known as copy number variations (CNVs). CNVs can be missed by gene sequencing, depending on the technology used, and can deleteriously disrupt gene expression and function. Both single nucleotide variations and deletion/duplication must be analyzed for overgrowth evaluation, either independently or using a technology that detects both in one assay. The tissue most often tested molecularly is the blood, in which DNA is extracted from circulating leukocytes, with the expectation that genetic changes leading to an overgrowth syndrome are found in a (pre-zygote) gamete and thus will be expressed uniformly. Genetic changes occurring in a stem cell in the post-zygote fetus will affect only the tissues developing from that cell, a phenomenon termed mosaicism, and only those tissues will harbor the genetic change. Mosaicism is discussed further below in the *segmental overgrowth* section where it is most common; however, it may occur in constitutional overgrowth as well (e.g., in BWS) and thus may complicate “traditional” molecular testing. The molecular testing include sequencing of genes of interest, CNV analysis, and epigenetic changes (abnormal DNA methylation), as discussed below.

For detecting CNVs involving genes responsible for overgrowth syndrome, comparative genomic hybridization (aCGH) has conventionally been utilized. In this technology, popularly referred to as chromosomal microarray analysis (CMA), a comparative assay is carried out between the genomic material of the patient and a standard reference based on its hybridization to a multiple fluorescent oligonucleotide fragments embedded to a chip. Any quantitative aberration from the standard represents a copy number variant up to a resolution determined by the embedded oligonucleotides. Dedicated multiple-gene panels have also been designed by commercial laboratories to detect both single nucleotide variations and small CNVs within the genes causing overgrowth syndromes on a single platform. In the 2000s, the commercialization of a sequencing technology termed next-generation sequencing (NGS) allowed massive parallel sequencing, enabling interrogation of hundreds and thousands of genes at one time. NGS sequencing is highly sensitive and can also identify mosaic variation with increasing sequencing depth, depending on the tissue tested. While a powerful tool, targeted panel NGS is limited by the number of genes that are covered in the assay. Whole-exome sequencing (WES) is an application of NGS that allows analysis of all protein coding genes across the human genome (23). WES is becoming the preferred testing strategy when differential diagnosis is broad. Although not utilized widely yet for overgrowth syndromes, evidence is emerging that WES as a first tier testing is a cost-effective approach in an increasing number of scenarios (24–27). Furthermore, with a newer technology of primers design, WES can provide adequate coverage of CNVs, i.e., genomic deletions and duplications (28, 29). It is important to note that targeted NGS panel, or WES in conjunction with CMA, will only detect aberrations in the specific tissue from which the DNA is extracted (saliva, blood, or from tissue biopsy) and will not detect epigenetic (methylation) changes. No single molecular testing covers all possible genetic changes related to overgrowth syndrome at this time. Any change in a gene from the published reference is considered to be a *variant*. Historically low-frequency variants (<1%) were referred to as mutation, and variants occurring at a higher frequency were referred to as polymorphism. The American College of Medical Genetics and Genomics (ACMG) classifies variants according to their effect on the gene product: (i) pathogenic, (ii) likely pathogenic, (iii) uncertain significance, (iv) likely benign, or (v) benign (30). Interpretation of a variant of uncertain significance can be difficult and is case dependent. Classification of variants is based on previously published cases, variant databases, prediction software, the nature of the change, and whether the variant is inherited or *de novo*. If a VUS is inherited from an unaffected parent, then it is more likely to be benign. Not surprisingly, if VUS is reported, parental samples may be needed for further investigation. It should also be noted that not all variants are deleterious, and association between variants and syndromes should be made carefully.

The focus of this article is to familiarize clinicians with some of the common genetic overgrowth syndromes caused by epigenetic and single-gene disorders.

## Overgrowth Syndromes Presenting Prenatally

### Beckwith–Wiedemann Spectrum: Beckwith–Wiedemann Syndrome and Hemihyperplasia

Beckwith–Wiedemann spectrum (BWSp; OMIM 130650) is the most common genetic overgrowth syndrome, with an estimated prevalence of 1/10,340 (31). It is classically seen with neonatal hypoglycemia, macroglossia, omphalocele, and/or visceromegaly. Prenatal history positive for polyhydramnios and prematurity is common. Other features include exophthalmos, slanted ear creases in the tragum and pits on the posterior helix, diastasis recti, facial nevus flammeus, inguinal or umbilical hernia, hyperplasia of the adrenal cortex, and occasionally congenital heart defect (32). Affected individuals are usually born macrosomic and develop rapid growth starting either at birth or before the first year of life. The accelerated linear growth plateaus around 8 years of age with final height in the 50–90% range for most individuals. Hemihyperplasia involving an extremity or face occurs in ~25% of BWSp individuals. Clinicians should note that asymmetry may not be apparent at birth, and overall symptoms may appear subtle. Macroglossia and hemihyperplasia, if present, tend to improve with time (33). Bone age is usually advanced, most notably in the first 4 years of life (34). Cardiomegaly and dome-shaped defect of the diaphragm can also be seen (33).

There is no consensus for the definition of this syndrome. It is currently viewed as a spectrum defined by three entities: *classical* BWS characterized by macroglossia, anterior abdominal wall defects, and prenatal and post-natal overgrowth; *isolated lateralized overgrowth* (previously *isolated hemihyperplasia*), and *atypical* BWS, in which patients exhibit the genetic abnormality associated with BWS but do not fit the two above clinically. The term BWSp encompasses all these categories underscoring the variability in phenotype due to mosaicism of genetic and epigenetic changes within 11p15.5.

**Figure 1:**
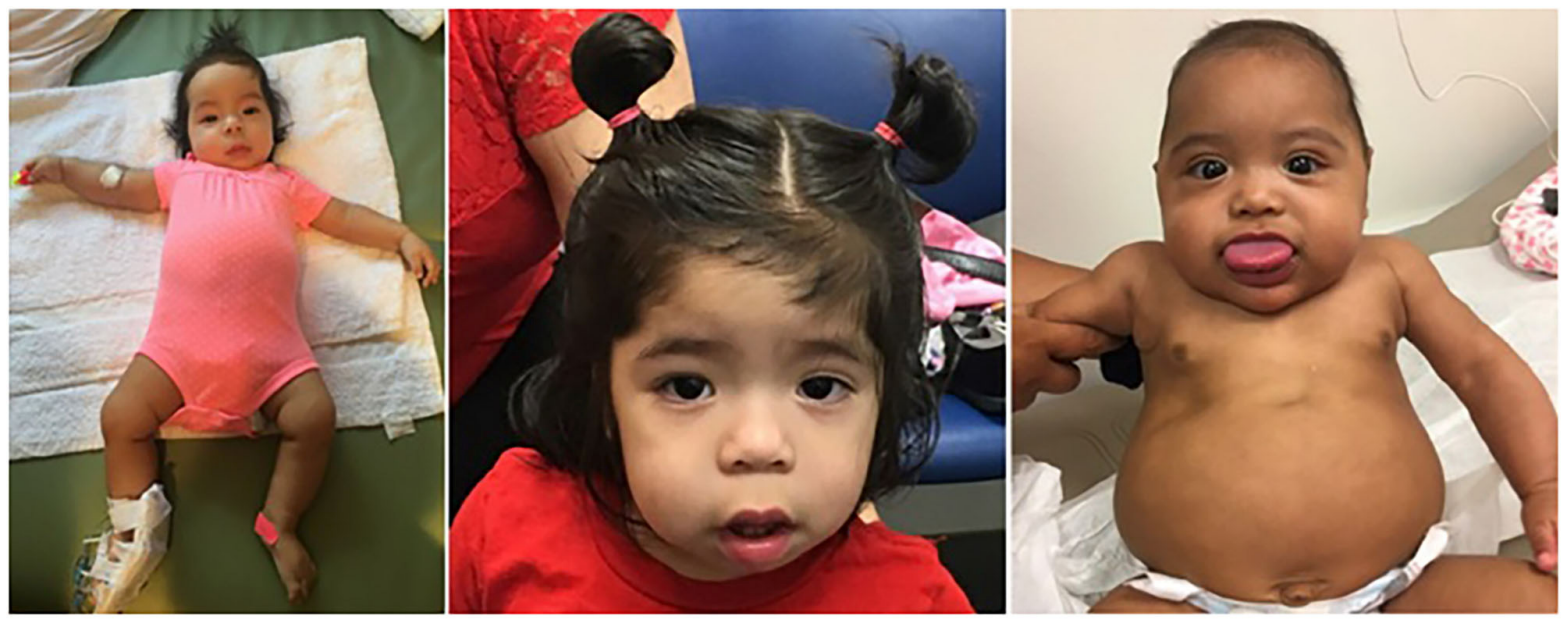
Two infants diagnosed with Beckwith–Wiedemann syndrome. The first infant (left and middle), presented with two episodes of symptomatic hypoglycemia, first occurring shortly after birth, and the second one at 7 months of age. Note the right-sided hemihyperplasia involving the right upper and lower extremities (1–1.5 cm difference in humeral and tibial circumferences, a 6% difference). Facial asymmetry was appreciated at 7 months, but can be readily seen in the middle, taken at 19 months of age. Extremity or facial asymmetry should raise suspicion for this syndrome. The patient in the right demonstrates the syndrome's most prominent feature, macroglossia. Methylation studies showed that the IC1 was hypermethylated: the paternal IC1 center is typically methylated, and maternal allele is not. Both patients undergo Beckwith–Wiedemann spectrum (BWSp)-specific cancer surveillance as depicted in Table 2.

In order to assist the clinician in making a diagnosis, the European Network for Congenital Imprinting Disorders established a scoring system (see Table 1). Existence of one cardinal feature such as macroglossia, lateralized growth, multifocal or bilateral Wilms tumor, or persistent hypoglycemia merits genetic testing, while 2 of these symptoms can establish a clinical diagnosis. Minor findings, like birthweight >2 SD, polyhydramnios, typical ear creases and/or pits, or typical BWSp tumors (most commonly Wilms tumor) also assist in making a diagnosis (35). For diagnostic purposes, two of the minor features are equivalent to one cardinal feature (and therefore merit genetic testing). Genetic testing is also recommended for patients with a family history of BWSp and a known heritable pathogenic anomaly. If a patient is suspected to have BWSp based on scoring but has a negative genetic testing, it is not unreasonable to refer to a BWSp expert for further evaluation (36). On the contrary, the presence of only one minor feature is inconsistent with BWSp (36). Figure 1 shows typical features of BWSp in two patients who are followed in our clinic.

**Table 1:** Clinical diagnostic criteria for Beckwith–Wiedemann syndrome.

**Feature**	**Pts**
**Cardinal findings**	
Macroglossia	2
Exomphalos	2
Lateralized overgrowth	2
Multifocal and/or bilateral Wilms tumor	2
Persistent hyperinsulinism (> 1 week)	2
Characteristic pathology: adrenal cortex cytomegaly, placental mesenchymal dysplasia, pancreatic adenomatosis	2
**Minor findings**	
Birthweight > 2 SD above the mean	1
Facial naevus simplex	1
Polyhydramnios	1
Ear creases and/or pits	1
Transient hyperinsulinism (<1 week)	1
Characteristic tumor: Unilateral Wilms tumor, neuroblastoma, rhabdomyosarcoma, hepatoblastoma, adrenocortical carcinoma, or pheochromocytoma	1
Nephromegaly and/or hepatomegaly	1
Umbilical hernia and/or diastasis recti	1
**Interpretation:**	
**Clinical score**	**Diagnosis**
4+	BWSp confirmed
2–3	Diagnosis by genetic testing
0–1	BWSp rejected

*Adapted from Brioude et al. (35)*.

**Table 2:** Types of overgrowth syndromes.

**Syndrome**	**Characteristics**	**Complications**	**Gene/locus**	**Tumor surveillance**
Perinatal overgrowth				
Beckwith–Wiedemann spectrum	Macroglossia, organomegaly, macrosomia, hypoglycemia, omphalocele, and hemihyperplasia	Wilms tumor, hepatoblastoma (see surveillance), rhabdomyosarcoma, and neuroblastoma Risk for BWSp is increased with assisted reproductive technology	11p15.5	Renal US including the adrenals every 3 months from diagnosis until the age of 7 + biannual physical examination; abdominal US every 3 months from diagnosis to the age of 4 + alpha-fetoprotein level
Simpson–Golabi–Behmel	Similar to BWSp, except that distinct facial dysmorphism become prominent with age, and nipples anomalies X linked disorder, seen primarily in males	Wilms tumor, hepatoblastoma (see surveillance), and neuroblastoma	*GPC3* (X-linked, Xq26.2)	Similar to BWSp; risk for malignancy is believed to be higher than the population risk
Sotos	Increased growth parameters, characteristic facial features, learning disabilities, and/or intellectual disabilities Tall stature and joint laxity may mimic Marfan syndrome	Seizures, neonatal jaundice, hypotonia, and cardiac anomalies; mild increased risk for Wilms tumor, hepatoblastoma, and neuroblastoma	*NSD1* (can result from small deletions on 5q35) *NFIX* for the similarly presenting Malan syndrome and *SETD2* for the similarly presenting Luscan–Lumish syndrome	None
Weaver	Similar to Sotos, except with excess loose skin and camptodactyly, and “Stuck-on” protruding chin	No increased malignancy risk Hypotonia, feeding difficulties, and ventriculomegaly	*EZH2*	None
*DNMT3A*-related	Similar to Sotos, except for round facies with thick eyebrows and prominent maxillary incisors. In contrast to Sotos, dysmorphic features increase with age	Seizures and cardiac anomalies	*DNMT3A*	None
Perlman	Macrosomia, macrocephaly, hypotonia, nephromegaly with nephroblastomatosis, abdominal wall weakness, and cryptorchidism	Post-natal mortality is ~87% Wilms tumor seen in about 1/3 of patients	*DIS3L2*	None
Segmental Overgrowth				
PTEN hamartoma tumor syndrome^*^	Macrocephaly, hamartomas, and intellectual disability BRR: pigmented macules on the penile shaft, and hamartomatous colonic polyps CS: trichilemmomas, papillomatous papules, and acral and plantar keratosis	Increased risk for breast, thyroid, renal, and endometrial carcinomas Colonic hamartomas may cause intussusception	*PTEN*	Breast—similar to BRCA 1/2 Endometrial—symptom based Colon—colonoscopy every 5 years starting at 35 years of age or 5–10 years prior to first familial case Thyroid—neck ultrasound at the age of 7 and then every 2 years Renal—ultrasound at 40 years of age and then every 1–2 years
PIK3CA-related segmental overgrowth^∧^	CLOVES—Lipomas, macrodactyly, scoliosis, body asymmetry, and skin wrinkling MCAP—megalencephaly, hypotonia, and limb asymmetry	Lipomas may cause cord compression, skeletal deformation Seizures	*PIK3CA* (overactivation)	None
KTS and PWS^%^	KTS: Asymmetric capillary/lymphatic malformations and limb overgrowth of usually the lower extremity PWS: similar but also characterized by arteriovenous fistulae	Varicosities, thrombophlebitis, pulmonary embolism. Arteriovenous fistulae may predispose to high output cardiac failure and distal arterial ischemia	*PIK3CA* (KTS) and *RASA1* (PWS)	None
Proteus syndrome	Extremely rare progressively deforming asymmetric overgrowth with characteristic cutaneous (cerebriform) connective tissue nevi. Most commonly affect distal lower limbs. Cranial hyperostosis, severe scoliosis, and vascular malformations are also common	Deep vein thrombosis and pulmonary embolism	*AKT1* (overactivation)	None

* *PTEN hamartoma tumor syndrome includes Cowden syndrome (CS), Bannayan–Riley–Ruvalcaba syndrome (BRB), and Proteus-like syndrome*.

∧ *PIK3CA-related segmental overgrowth includes two distinct phenotypes: CLOVES, congenital lipomatous overgrowth, vascular malformations, epidermal naevi, scoliosis/skeletal, and spinal syndrome; MCAP, megalencephaly–capillary malformation*.

% *KTS, Klippel–Trenaunay syndrome; PWS, Parkes–Weber syndrome*.

**Figure 2:**
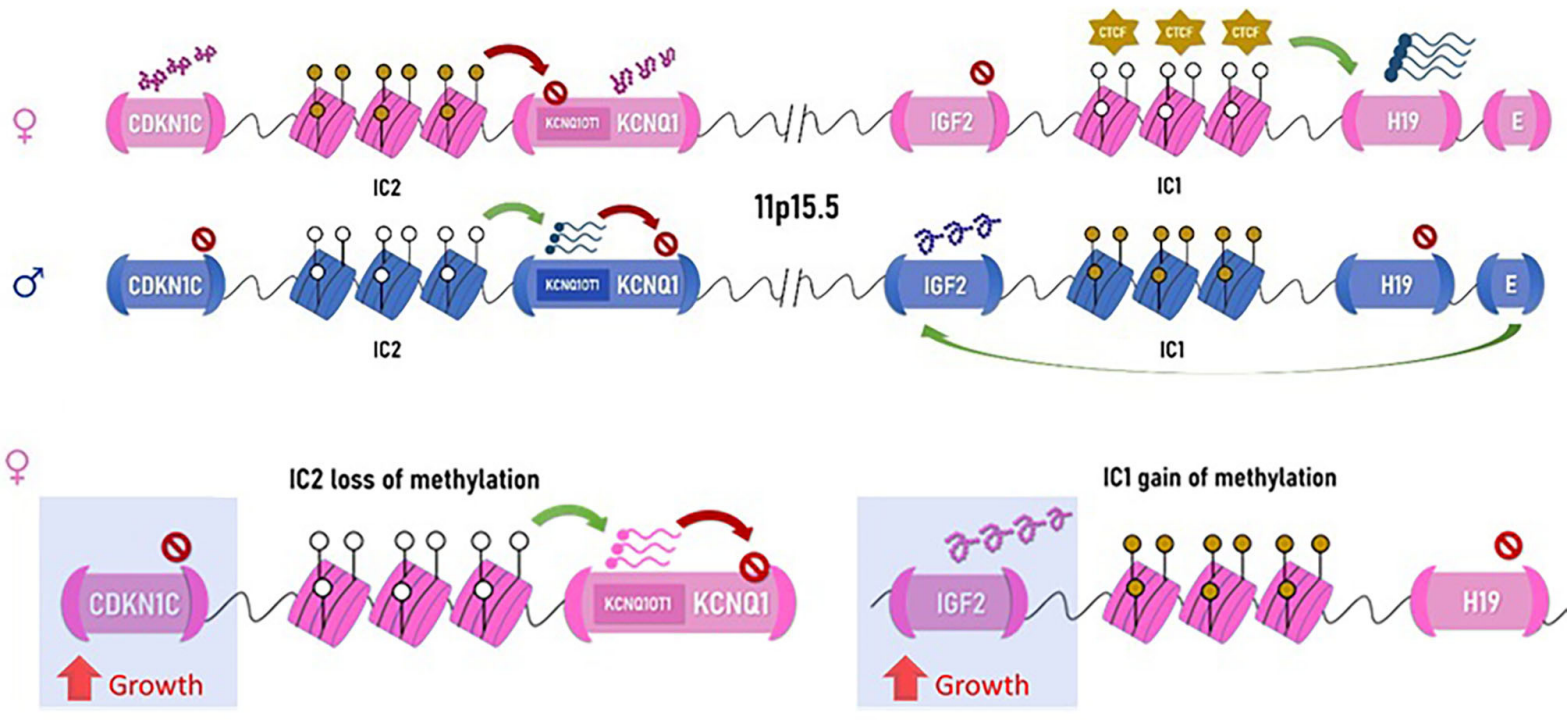
Chromosomal arrangement of the 11p15.5 locus. Maternal allele on top (in pink) and paternal allele below (blue) are represented. IC1, imprinting center 1, attracts the non-methylated form CTCF (transcription repressors of the CCCTC-binding family), which activates transcription of H19, a non-coding RNA, which represses growth. When the imprinting center is methylated, as normally occurring on the paternal allele, H19 is not transcribed, and the downstream enhancer elements can act on IGF2, which similar to IGF-1, promotes growth, particularly in the perinatal phase. Imprinting center 2 represses the expression of the potassium channel gene, *KCNQ1*, via transcription of its antisense (KCNQ1OT1) and the nearby *CDKN1C*, a growth-repressing cycline. Therefore, when IC2 is methylated, as seen on the maternal allele, CDKN1C is expressed, and growth is attenuated. On the paternal allele, IC2 is not methylated, and CDKN1C along with KCNQ1 are repressed, allowing growth. Either via expression of IGF2 or silencing of CDKN1C, the paternal allele promotes growth. Hypermethylation of IC1 on the maternal allele resulting in IGF2 overexpression is the mechanism seen in the patient in Figure 1 (right). This causes the maternal allele to function similar to the paternal allele, resulting in overgrowth with macroglossia. Loss of methylation of the maternal IC2 resulting in CDKN1C repression will also result in BWSp. Note: IC2 is depicted in this figure in juxtaposition to the KCNQ1 gene for simplification; its true position is within the KCNQ1 gene.

The syndrome is a complex multigenic disorder caused by modifications of growth regulatory elements on 11p15.5 [short arm [p] of chromosome 11, region 1, segment 5, subsegment 5], which can explain the phenotypic variability (33, 36). The molecular arrangement of 11p15.5 locus demonstrates concerted epigenetic regulation of gene expression: It contains two imprinting centers (IC1 and IC2) that are responsible for silencing maternally or paternally inherited gene expression by DNA methylation (see Figure 2). IC1 is methylated on the paternal allele (the copy inherited from the father), suppressing the expression of nearby *H19* gene, a non-coding RNA (ncRNA), which negatively regulates growth. Another nearby gene, *IGF2*, is then freely transcribed and promotes somatic growth. IC1 is not methylated on the maternal allele, and the opposite is seen: transcription factors bind IC1, remodel the chromatin, and transcribe *H19*, leaving *IGF2* promoter insulated from its enhancers and thus repressed (37). Overexpression of IGF2 can occur either by gain of methylation at IC1 on the maternal allele, duplication of the paternal allele, a variant causing inactivation of IC1 on the maternal allele, or uniparental disomy (UPD) in which the offspring inherits two paternal copies of 11p15.5 instead of one paternal and one maternal copy. Such overexpression can lead to BWSp features; IGF2 overexpression is also seen in 70% of Wilms tumor (38), explaining the increased predisposition to Wilms tumor in BWSp patients. IC2 promotes the expression of *KCNQ1OT1* gene and is methylated on the maternal allele. *KCNQ1OT1* is an antisense of *KCNQ1* gene, and therefore, its expression inhibits the expression *KCNQ1*. Since IC2 is methylated on the maternal allele, *KCNQ1OT1* is not expressed, and thus the potassium channel *KCNQ1* along with the nearby *CDKN1C* gene are both expressed on the maternal copy. The opposite is seen on the paternal allele. Loss of methylation of IC2 on the maternal allele or inactivating variants of *CDKN1C* (in an autosomal dominant fashion) both lead to decreased expression of the *CDKN1C* gene from the maternal allele (because of the *KCNQ1OT1* antisense) in BWSp patients. *CDKN1C* is a cyclin-dependent kinase, which negatively regulates growth, and its overexpression is found in a few growth retardation syndromes (39).

A tier-based algorithm for molecular testing has been established when molecular testing is indicated (i.e., at least one major criterion or two minor criteria are present) (36, 40). The first tier consists of methylation study, which is abnormal in about 75% of patients with BWSp. Methylation study can unveil multiple mechanisms including gain of methylation on IC1, loss of methylation on IC2, or both (which is, in essence, paternal UPD); it will also be abnormal with a loss of chromosomal segment on 11p15.5 (along with its proper methylation). Methylation-specific multiplex ligation-dependent probe amplification (MS-MLPA) can detect epigenetic abnormalities as well as identify CNVs of the 11p15 region. If this study is abnormal, then SNP-based array should be considered to identify both CNVs within 11p15.5 and pUPD, which is observed in ~20% of BWSp. If the methylation study is negative, sequence analysis of *CDKN1C* for loss of function on the maternal allele is warranted (41). Variants in *CDKN1C* can be seen in ~5% of the affected individuals, increasing to ~40% in familial cases of BWSp (which are 15% of all BWSp cases) (42). When all testing is negative, as seen in ~20% of BWSp individuals, sampling tissue other than blood—saliva, skin, or directly from a hypertrophied tissue, may identify low-level mosaic genetic and epigenetic changes within 11p15.5 in up to 10% of affected patients (35, 36).

The importance of the specific genetic diagnosis is for assessment of recurrence risk in families: methylation abnormalities in the absence of small deletions/duplications are associated with sporadic cases of BWSp and the recurrence risk is estimated at <1%, while *CDKN1C* mutation carries a recurrence risk of 50% (inherited as an autosomal dominant trait from maternal transmission). It is recommended that if a *CDKN1C* variant is ascertained, the mother and other family members should also be referred for genetic evaluation, as BWSp can manifest with subtle clinical expression.

Contrary to previously held perception, the neurocognitive development of BWSp patients is similar to the general population, and therefore no additional surveillance is recommended for development. Comorbidities such as prematurity, present in 34–41%, or hypoglycemia, seen in 30–50%, may affect normal development (42, 43). If an early diagnosis of BWSp is made, it is important to monitor glucose levels similarly to babies of diabetic mothers. For the same reason as mentioned above, newborn siblings of affected individuals should also be monitored for hypoglycemia even in the absence of classic symptoms.

The importance of early diagnosis of BWSp stems from an increased risk for several tumors. The overall risk of malignancy among BWSp patients is estimated at 7.5%: 4% Wilms tumor, 1% hepatoblastoma (relative risk of 2,280), 0.5% rhabdomyosarcoma, 0.5% neuroblastoma, and ~1.3% all other tumors (of note is adrenocortical tumor) (35, 40, 41). Interestingly, in 3% of isolated Wilms tumor, constitutional genetic changes in 11p15.5 are seen (a locus different than the WT1 gene) (44). In BWSp, the increased propensity is mostly seen for so-called *embryonal tumors* which develop early in life: hepatoblastoma and neuroblastoma develop by the age of 2 and Wilms tumor prior to the age of 8 (45). Less data are available for isolated lateralized overgrowth (isolated hemihyperplasia), but neoplasia incidence is reported as high as 6% (46). The diverse molecular mechanism of BWSp intrigued researchers to differentiate tumor risk and surveillance based on molecular background [e.g., Maas et al. (45) and Brioude et al. (40)]. Gain of methylation on IC1 is associated with an increased risk for embryonal tumors (28%), mostly Wilms tumor, as opposed to loss of methylation on IC2, which has a much lower risk of tumors of about 2.6% with a higher propensity to develop hepatoblastoma; *CDKN1C* variations and pUPD11 impart an intermediate tumor risk, ranging from ~7–16%, respectively (36). The international consensus statement recommend genotype-based tumor screening (35); however, genotype-based stratification is still debated (47, 48) and current guidelines of the American Association of Cancer Research (AACR) recommend uniform surveillance for all syndromes with an increased propensity for Wilms tumor and hepatoblastoma (49): renal ultrasound (US) including the adrenals every 3 months from diagnosis until the age of 7 for early detection of WT (and adrenal malignancy) along with biannual physical examination; for hepatoblastoma, abdominal US every 3 months from diagnosis to the age of 4 years along with serum alpha-fetoprotein for distinguishing hepatoblastoma from hemangioma. It should be noted that BWSp patients tend to have higher levels of alpha-fetoprotein (AFP) than the general population (36), and proper surveillance should rely on serial measurements rather than a threshold value. The interpretation difficulties have led some experts to dispute the utility of measuring AFP for tumor surveillance (50). AFP testing is currently lacking in the international consensus statement (35). No specific surveillance is provided for rhabdomyosarcoma; however, the serial abdominal US recommended for the first 4 years of life can assist in early detection of rhabdomyosarcoma as well. A longer renal surveillance by US may be warranted if unilateral or bilateral nephromegaly, cystic changes, or duplication of the collecting system are seen, which are frequent in BWSp. Despite the common finding of macroglossia (80%), surgical intervention is only rarely indicated (51) in cases associated with symptoms of respiratory problems, obstructive sleep apnea, feeding difficulties, persistent drooling, problems with speech and articulation, and orthodontic problems (36).

BWSp, along with other imprinting disorders, is observed to occur at higher frequencies among couples utilizing assisted reproductive technologies (ART). The excess risk in two European cohorts was demonstrated to be as high as 3–10-fold (43, 52). In the United States, a study found a 20-fold overrepresentation of IVF cases in fetuses diagnosed with BWSp by omphalocele (53). Meta-analysis from 2018 found a pooled OR of 5.8 from eight different studies (54). The absolute risk, however, remains relatively low (<1 in 1,000) (35). The mechanism behind this observation is currently not clear. Couples utilizing ART should therefore be informed about the increased risk.

Prenatally, suspected sonographic findings for BWSp include long umbilical cord, placentomegaly, abdominal wall defect, nephromegaly, and cysts of adrenal glands. Methylation studies of cells obtained by amniocentesis and chorionic villi sampling (CVS) can assist in early confirmation of the diagnosis (41, 55). Suspected or confirmed prenatal BWSp diagnosis should warrant a delivery in a high risk unit because of the increased risk for hypoglycemia, fetal macrosomia, omphalocele, and macroglossia. In addition, abdominal imaging should be obtained postnatally. Clinicians should maintain a high index of suspicion for cardiac anomalies as well. A strong clinical suspicion should guide the management of the patients even with a negative genetic test (see Table 1). We refer the readers to a comprehensive review (36) and to the international consensus statement (35) regarding this relatively common overgrowth syndrome with a wide phenotypical spectrum and a complex epigenetic makeup.

### Simpson–Golabi–Behmel Syndrome

Simpson–Golabi–Behmel (SGB) syndrome (OMIM 312870) is an X-linked prenatal and post-natal overgrowth syndrome associated with characteristic dysmorphic features. It affects primarily males and is associated with loss-of-function variants in the growth modulator proteoglycan, *GPC3* on Xq26.2. Affected individuals typically exhibit increase in all growth parameters (>97% in length, weight, and head circumference). Macrocephaly is reported in 70% of cases; other common features include ocular hypertelorism (wide-spaced eyes) with broad upturned nose, macroglossia, and macrostomia (large mouth), supernumerary nipples, pectus excavatum, and hypotonia. Less common features include congenital heart defect (seen in ~36%), polydactyly with nail hypoplasia, dental malocclusion, rib anomalies, cleft lip or palate (observed in ~13%) visceromegaly, umbilical hernia, and genitourinary anomalies (cryptorchidism, gonadal dysgenesis) (56).

To a pediatrician, a newborn with SGB may present similarly to BWSp: macrosomia, macroglossia, visceromegaly, and umbilical hernia. Bone age is advanced. To complicate it further, polyhydramnios, prematurity are common as well, and in 26% of cases, neonatal hypoglycemia is seen (57). However, several clues can guide the correct diagnosis— SGB patients appear more dysmorphic with musculoskeletal abnormalities and nipple abnormalities (supernumerary or bifid). Abdominal wall defects such as omphalocele are generally not observed. Another clue is that the degree of dysmorphism increases with age in SGB, while the opposite is true for BWSp. Since SGB is X-linked, the majority of affected individuals are males, with females presenting with milder symptoms. While rare, a “full-blown” phenotype has been reported in few females (58). Development in SGB is reportedly normal.

Similarly to BWSp, SGB patients are reported to have an increased risk of childhood malignancy including Wilms tumor, hepatoblastoma, and adrenal neuroblastoma. The risk is estimated at 10%; however, the relative risk has not been established (59). Surveillance recommendations are therefore similar to BWSp.

### Sotos Syndrome

Sotos syndrome (OMIM 117550), previously referred to as cerebral gigantism, is an overgrowth syndrome characterized by a triad of (i) overgrowth (increased height, macrosomia, and macrocephaly) (ii) characteristic facial features, and (iii) learning disabilities and intellectual disabilities. Newborns have tall stature; the majority are >99%, owing to disproportionally long limbs; compared to BWSp and SBG syndromes, the “average” Sotos patient is taller. Bone age is also advanced; however, as seen with many other overgrowth syndromes after the first 4 years of life, the accelerated growth plateaus with the final height reaching approximately in the 90th centile. Classic facial characteristics include frontal bossing, dolichocephaly (elongated occipito-frontal axis) and fronto-parietal balding seen in >90% of patients. Early eruption of deciduous teeth and high arched palate are common as well. Learning disability can be seen in 97% of patients, along with intellectual disability, ranging from mild to severe. However, these data may represent ascertainment bias as normally functioning children may not be referred for evaluation. A study of a cohort of 52 patients with Sotos syndrome found an IQ score of 61 with SD of 17 (60). Hypotonia, seen in 70% of patients, is believed to contribute to motor delays, expressed as difficulty with early feeding and walking (after 15 months). Hypotonia tends to improve with age, however delays in expressive language are frequently seen. The cognitive profile of Sotos syndrome patients typically shows strength in verbal ability and visuospatial memory but relative weakness in non-verbal reasoning ability and quantitative reasoning. Seizures have been reported in 9–50%, of which about half develop epilepsy (61). Other findings include neonatal jaundice (seen in 75%), cardiac anomalies (20%), maternal pre-eclampsia (17%), renal anomalies (15%), joint laxity, and scoliosis (15%). Obesity is rare, with 74% of patients showing BMI below the 95% (62).

Sotos syndrome is an autosomal dominant disorder caused by mutations in the nuclear receptor SET domain-containing protein 1 (*NSD1*) gene located on 5q35. It encodes a histone methyltransferase but with an unknown function. The overall prevalence of Sotos syndrome is estimated at 1 in 14,000. More than 95% of the cases arise from *de novo* mutations (resulting from a mutation in a gamete of a parent); however, few cases of familial Sotos syndrome have been reported. Most mutations causing Sotos syndrome are point mutations—changes in the sequence that substitute one amino acid with another (missense variant) or causing early termination of transcription (non-sense). About 9% of individuals with Sotos syndrome of European ancestry and ~50% of those of Japanese ancestry have a specific deletion of the chromosomal region flanking *NSD1* gene. This common deletion arises from the unique chromosomal structure of the 5q35 region: NSD1 gene is flanked by two regions of a repetitive sequence (called LCR, low-copy repeats). When the chromosomes are aligned for recombination in the formation of gametes, the DNA replication machinery may align the proximal LCR region on one chromosome with the distal LCR region on the other homologous chromosome and DNA replication will create a chromosome missing the region in the middle, including the NSD1 and other flanking genes. Deletions and duplications created by misalignment of repetitive sequences during recombination is an important mechanism of genetic diseases.

Malignancy rate is reportedly low, with sporadic reports of neuroblastoma, teratomas, and leukemia; no specific tumor surveillance is recommended. Long limbs, joint laxity, and scoliosis can be confused with Marfan syndrome, and the macrocephaly and learning disabilities can lead to a consideration of fragile X syndrome. Clinicians should therefore keep Sotos syndrome in mind when evaluating such patients (33, 60, 61, 63–66).

Sotos syndrome is also the most common syndrome within the overgrowth with intellectual disability (OGID) disorders (67). Other syndromes in this category include Weaver syndrome and DNMT3A-related OGID (see below). Both Sotos and Weaver syndromes may present with mild degree of dysmorphism; primary clinicians should consider OGID in a patient that appears large for chronological age and presents with developmental delay and/or intellectual disability but without obvious dysmorphism.

Affected individuals who appear to have Sotos syndrome based on clinical evaluation but with negative genetic testing, may, in fact, have Malan syndrome, previously called Sotos syndrome 2 (OMIM 614753). It is associated with mutations in the *NFIX* gene on 19p13.13. Patients with either Sotos or Malan syndrome demonstrate accelerated initial growth that plateaus later in life, share facial dysmorphism (long face and prominent forehead, down slanting palpebral fissures), marfanoid body habitus (slender appearance with long upper extremities), hypotonia, and intellectual disability/learning disabilities. However, other facial features may differ. In addition, Malan patients exhibit myopia, while Sotos syndrome patients typically exhibit hyperopia. Anxiety is a feature that is more common among Malan syndrome patients than Sotos (68). Seizures, like in Sotos syndrome, are reported in a minority of patients with mutations in *NFIX* (about 20%). However, with contiguous gene deletion of 300 kilobases to 3 megabases at the 19p13.13 locus that includes *NFIX* and *CACNA1A*, also known as the 19p13.13 microdeletion syndrome (OMIM 613638), seizures occur frequently; the deletion of *NFIX* results in overgrowth and the loss of the voltage-gated calcium channel *CACNA1A* causes seizures. Therefore, the cause of overgrowth with intractable seizures in these individuals may be detected by chromosomal microarray, which can readily detect such genomic deletions, but not by single-gene sequencing. Variants in *NFIX* can also cause Marshall–Smith syndrome (OMIM 602535), a syndrome of advanced bone age and increased length at birth but associated with failure to thrive, small chin, proptosis (protrusion of the globus), blue sclerae, and underdevelopment of the malar area of the face. Malan and Marshall–Smith syndromes are called *allelic* to each other, arising from variants in the same gene but resulting in two distinct syndromes (68, 69).

*SETD2*-related disorder is another example of a Sotos-like autosomal dominant overgrowth syndrome resulting in post-natal overgrowth, macrocephaly, prominent forehead, and advanced bone age, named Luscan–Lumish syndrome (OMIM 616831), (70), adding another layer of complexity to a diagnosis of Sotos syndrome based on clinical symptoms alone.

### Weaver Syndrome

**Figure 3:**
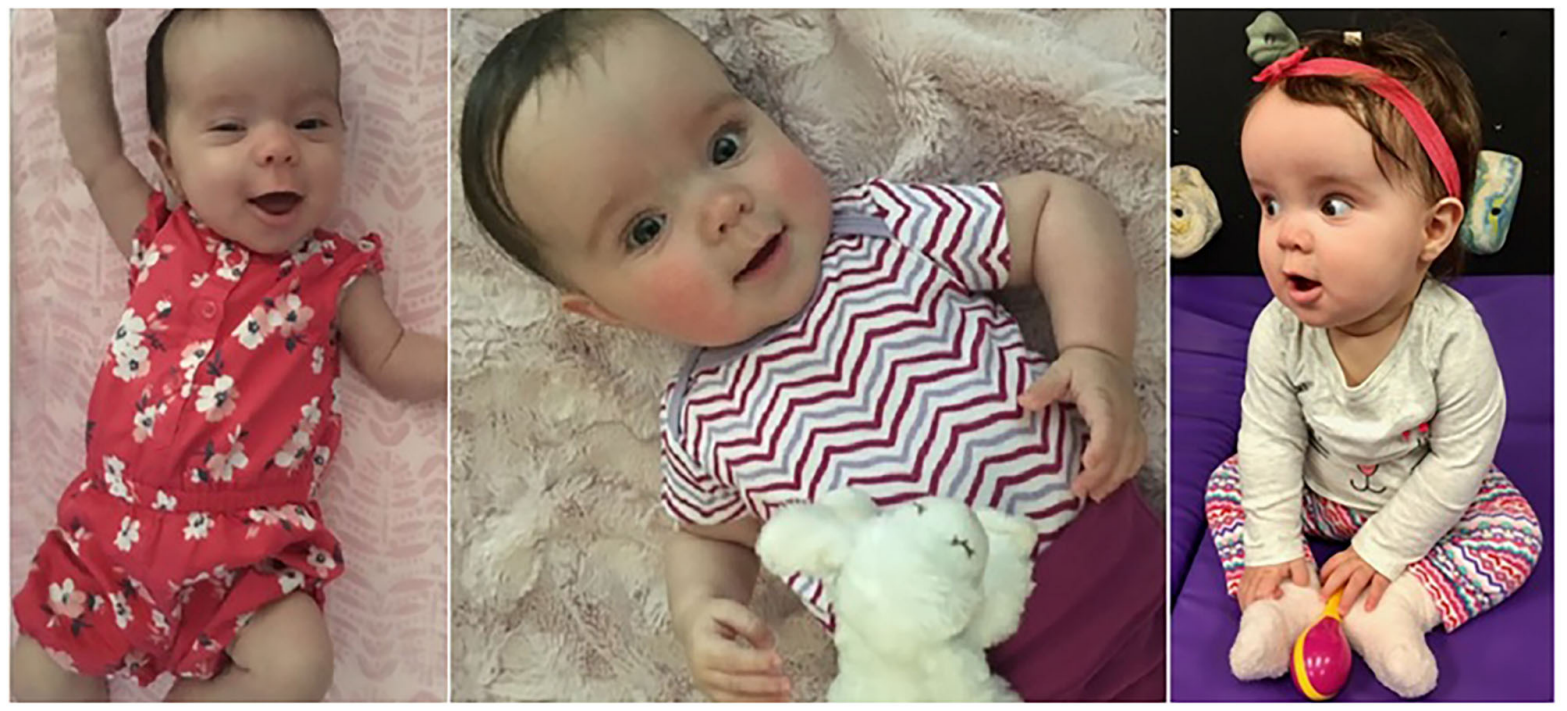
A patient diagnosed with phosphatase and tensin homolog (PTEN)-hamartoma tumor syndrome. She was brought to medical attention shortly after birth for concerns of macrocephaly and hypotonia. Her brain MRI was normal. At 14 months, her fronto-occipital circumference (FOC) was 52.6 cm (+5.38 SD) and 56.4 cm (+5.06 SD) at 35 months. She has been receiving physical therapy since age 6 months due to hypotonia and also speech therapy for expressive language delays. FOC > 3 SD *even as isolated finding* is suspicious of PTEN-hamartoma tumor syndrome. Due to the increased risk for malignancy (see text and Table 2), she will undergo childhood cancer screening (thyroid carcinoma) and later adult cancer screening (breast, thyroid, endometrial, and colon). Developmental delays are common in this syndrome.

Weaver syndrome (OMIM 277590) is an accelerated growth syndrome defined by increased height and weight (>2D), advanced bone age, broad forehead with flat occiput, excess loose skin, camptodactyly [bent finger(s) due to proximal interphalangeal joint abnormality] and variable degree of intellectual disability (seen in 80% of patients). Characteristic facial appearance includes hypertelorism (widely spaced eyes), large ears, and a “stuck on” protruding chin. Newborns and infants often have truncal hypotonia (~50%) and extremity hypertonia with limited elbow extension. Hypotonia is associated with poor feeding and may also be associated with a characteristic weak, low pitched cry affecting about 50% of patients. Developmental delays are common, both in gross motor (sitting at ~10 months, walking at 15–20 months) and fine motor skills (poor coordination). Ventriculomegaly is frequently seen on brain imaging. Advanced bone age is uniformly reported, exceeding the chronological age by a factor of 1.5–2 (71). The triad of post-natal overgrowth, advanced bone age, and intellectual disability especially in the setting of mild facial dysmorphism can make a distinction between Weaver and Sotos syndromes difficult. To complicate it further, the characteristic features tend to improve with age in Weaver syndrome. Genetic testing can assist in establishing a diagnosis: The genetic change associated with Weaver syndrome is in *EZH2*, a histone methyltransferase, which is an important component of the highly conserved repression complex PRC2 (polycomb repressive complex 2). It is inherited in an autosomal dominant manner. Interestingly, pathogenic variants in *EZH2* have been found in individuals with overgrowth without other clinical features of Weaver syndrome, indicating a wide spectrum of the disorder (72). There is no evidence to link increased risk of malignancy with Weaver syndrome to date, and therefore there are no specific cancer screening recommendations (33, 71–73). PRC2 is comprised of three core subunits, encoded by the genes *EZH2, SUZ12*, and *EED*. Variants in the latter two components are known to cause Weaver-like syndrome (WLS, OMIM 618786) (74, 75) and Cohen–Gibson syndrome (COGIS, OMIM 617561) (76), respectively. These two pre- and post-natal overgrowth syndromes are significantly rarer than WS, yet they share similarities that include accelerated bone maturation, hypertelorism, large ears, umbilical hernia, stuck-on chin, and non-specific changes on brain MRI. COGIS also shares intellectual disability and small and retracted mandible (microretrognathia); however, scoliosis, undescended testes (cryptorchidism), and cardiac defects are significantly more common than in WS (77). Figure 3, adapted from (78), depicts a patient with typical Weaver syndrome feature who was found to have a variant in *EED* gene. WLS, the rarest of the autosomal dominant PRC2-related overgrowth syndrome, does not include the characteristic camptodactyly, abnormal tone (either hypertonia or hypotonia) and microretrognathia seen in WS. Intellectual disability has been reported in one out of three published cases (74). In our institution, we have encountered an adolescent and an adult (the mother of the adolescent) who were diagnosed with WLS. Both have normal intellect, prenatal overgrowth, and both presented with malignant bone tumors.

### *DNMT3A*-Related Overgrowth Syndrome

Another autosomal dominant overgrowth and intellectual disability (OGID) syndrome, similar to Sotos and Weaver syndromes, is a DNMT3A-related overgrowth syndrome, also known as Tatton–Brown–Rahman syndrome (OMIM 615879). It is caused by pathogenic variants in DNA methyltransferase 3A. Despite sharing a similar mechanism with Weaver syndrome, that is, a heterozygote mutation (pathogenic variant in one copy) in DNA methyltransferase, this syndrome bears more similarities to Sotos syndrome. Both include features of macrocephaly noticed at birth, joint hyperlaxity, scoliosis, hypotonia, and seizures. However, the facial dysmorphism is different and includes round facies, straight and thick (bushy) eyebrows, and prominent maxillary incisors. These features evolve over time and may not be present in early childhood. Moreover, unlike Sotos and Weaver syndromes, patients' length at birth is usually normal and increases to >2 SD only later in life. About 16% of individuals are reported to have congenital heart defects; therefore, an echocardiography should be considered upon diagnosis (79).

Compared with the Sotos and Weaver syndromes, the “average patient” is more intellectually impaired. A cohort of 55 patients from 41 families (79) found that 82% were diagnosed with moderate to severe intellectual disability, compared with 28% in Weaver syndrome (74) and 43% in Sotos syndrome (80). Furthermore, 36% of affected individuals were diagnosed with autism.

Non-congenital or somatic variants in *DNMT3A* can be found in patients with acute myeloid leukemia (AML), myelodysplastic syndrome, and myeloproliferative syndromes. Interestingly, 26 out of the 40 variants described (63%) in *DNMT3A*, (79) were also found in patients with AML according to the Catalog of Somatic Mutations in Cancer (COSMIC) database (81). In particular, missense mutations in arginine at position 882 causing a change either to cysteine or histidine (a change seen in about half of the cases of AML with a mutation in *DNMT3A*) were also documented in affected patients with the overgrowth syndrome. Two out of the reported 77 patients with this OGID syndrome developed AML at the ages of 12 and 15 (79); however, despite the perceived increase in the odds ratio, conclusions cannot be drawn for an actual increased risk due to the small number of patients. Similarly, in Weaver syndrome, non-congenital *overexpression* of the DNA methyltransferase *EZH2* is found in several solid tumors and non-Hodgkin lymphoma, a change that serves as a negative prognostic factor (82), yet germline variants in this gene causing Weaver syndrome are not linked to an increased risk of those tumors.

### Perlman Syndrome

Perlman syndrome (OMIM 267000) is a syndrome characterized by macrosomia, macrocephaly, round facies, hypotonia, and visceromegaly. Visceromegaly most commonly involves the kidneys (nephromegaly) or liver (hepatomegaly) but can also include the heart, spleen, and pancreatic islet cells (leading to neonatal hypoglycemia). Nephromegaly, seen in 80–100% of affected individuals, is frequently accompanied by nephroblastomatosis (diffuse persistence of metanephric blastema), a characteristic finding in Perlman syndrome, that predisposes to Wilms tumor seen in about a third of the patients. Other common findings are cryptorchidism and inguinal hernias. Newborns commonly present with abdominal distention as a result of nephromegaly, hepatomegaly, ascites, and/or abdominal wall muscular hypoplasia. The abdominal distension induces, in turn, hypoplastic lungs. About 87% of the affected infants develop respiratory distress and/or renal failure and die within the first hours or days of life. In surviving patients, growth parameters typically decline rapidly to reach the lower end of normal.

Prenatal diagnosis can be suggested based on nephromegaly, polyhydramnios, and fetal ascites (33, 83). It may be very difficult to differentiate Perlman syndrome from the other overgrowth syndrome mentioned above and thus genetic testing is an important part of the evaluation. Perlman syndrome is an autosomal recessive syndrome due to mutation in the gene encoding *DIS3L2* exoribonuclease (RNAase); *DIS3L2* has been shown to lead to *IGF2* overexpression, as seen in the Beckwith–Wiedemann syndrome and is strongly associated with tissue overgrowth and Wilms tumor development (84).

## Segmental Overgrowth Syndromes

### Proteus Syndrome

Proteus syndrome (OMIM 176920) is a complex syndrome with variable presentation consisting of progressive segmental overgrowth of the feet and/or hands, cutaneous connective tissue nevi, and cranial hyperostosis. Affected individuals have minimal manifestation at birth and are born with normal growth parameters. Starting around 6–18 months of age, they can develop excessive asymmetric growth that can reach twice its normal size by age 6 years. The growth is of the bones and the soft tissue, and although the hands and feet are most commonly involved, any bone can be affected. Leg length discrepancy of up to 20 cm and scoliosis of more than 90° have been reported. Final height is normal as skeletal growth plateaus at adolescence. In childhood, patients can develop cutaneous connective tissue nevi most commonly involving the feet, hands, abdomen, or nose. These nevi are highly collagenized connective tissues that are firm with their surface resembling the gyri and sulci of the brain, thus named cerebriform. These nevi are pathognomonic for Proteus syndrome however they should be differentiated from the neurofibromas seen in neurofibromatosis syndrome; neurofibromas are smooth, soft, movable, and rarely involve the plantar aspect of the foot. Another cutaneous finding seen in Proteus syndrome is epidermal nevi. These lesions can present as early as the first few months of life and tend to follow the lines of Blaschko. Adipose tissue overgrowth is common in this syndrome; focal growth tends to occur from early infancy to early adulthood. They are not encapsulated lipomas but their histology is almost always benign. Along with growth, there are areas of adipose atrophy giving a combined presentation of adipose dysregulation. Skeletal abnormalities can occur anywhere in the body and add further to the striking asymmetry. Restrictive pulmonary disease is therefore common. Other distinctive features of Proteus syndrome include cranial hyperostosis, condylar (mandibular) hyperplasia, and rarely craniosynostosis (33, 85, 86).

Vascular anomalies are common in Proteus syndrome and include lymphatic, venous, and capillary vessels. They can be recognized in the first few months of life and tend to grow with the patient, or expand, but generally do not regress. Deep vein thrombosis and pulmonary embolism (PE) complicates these vascular anomalies and can lead to early death.

The majority of individuals with Proteus syndrome have normal intelligence; however, a subgroup (30%) of patients with dysmorphic facial features has seizures and intellectual disability. Dysmorphism includes dolichocephaly (elongated sagittal axis), long face, downslanting of the palpebral fissures (the long axis of the eye is slanted down temporally), and open mouth at rest. Other organs that are commonly affected include the eye (strabismus, nystagmus, myopia, and retinal detachment), lungs (13% with cystic changes), and kidneys (nephrogenic diabetes mellitus, renal cysts, heminephromegaly, duplications of the renal collecting system, and hydronephrosis) (33). It has been observed that Proteus syndrome predisposes to a wide variety of tumors. Rarity of the syndrome has limited recommendations about specific tumor surveillance strategy. Two specific tumor types, monomorphic adenomas of the parotid glands and bilateral ovarian cystadenomas are specific enough to assist in making a diagnosis of Proteus syndrome, but the data are insufficient to show that early detection could change outcome (87).

To date, only one mutation in one gene has been linked to Proteus syndrome. A change from glutamate to lysine at position 17 results in overactivation of the *AKT1* gene, which is part of the PI3KA/AKT1/mTor proliferation pathway (88). Not surprisingly, molecular testing for *AKT1* detects the variant in only 47% of Proteus syndrome cases (85)—when possible, it is preferable to obtain biopsy from an affected tissue to increase the detection yield.

Due to the variable expressivity of this syndrome, diagnostic criteria have been suggested and include the key features of this syndrome including mosaic distribution of lesions, sporadic (non-inheritable) occurrence, and progressive course. Lesions can be either the pathognomonic cutaneous (cerebriform) connective tissue nevi, or other findings such as asymmetric growth, dysregulated adipose tissue growth, lung cysts, and/or the specific tumors (monomorphic adenomas of the parotid glands and bilateral ovarian cystadenomas). These criteria were constructed to avoid over-diagnosis of this syndrome. A review of 205 published cases of Proteus syndrome found that only 95 of them fulfilled these criteria, and 80 cases (39%) “clearly did not” (86). It is recommended to complement clinical diagnosis with molecular testing. The incidence of the syndrome is estimated to be 1 in a million to 10 million (85). It is therefore recommended to first consider other relatively frequently seen diagnoses such as neurofibromatosis type 1, when encountering disfiguring cutaneous lesions that could represent plexiform neurofibromas. Additionally, if the segmental overgrowth is presented soon after birth, Proteus syndrome may be less likely.

### Phosphatase and Tensin Homolog Hamartoma Tumor Syndrome

Phosphatase and tensin homolog (PTEN) Hamartoma tumor syndrome (PHTS) is a disorder encompassing three seemingly distinct clinical syndromes—Cowden syndrome, Bannayan–Riley–Ruvalcaba syndrome, and Proteus-like syndrome—all of which are characterized by mutations in the tumor suppressor gene *PTEN* (Phosphatase and tensin homolog)—characterized by unregulated cellular proliferation leading to the formation of hamartomas. PTEN is a phosphatase that removes a phosphate from the second messenger phosphatidylinositol triphosphate and, by doing so, inhibits the Akt (Protein kinase B) pathway, a cardinal pathway of cell proliferation and angiogenesis. Not surprisingly, somatic inactivating mutations in *PTEN* are found in breast, prostate, lung, endometrial carcinomas and glioblastoma. In cancer, *PTEN* mutations are acquired in adulthood in a single cell which proliferates to create a tumor (“somatic” mutations). Alternatively, *PTEN* mutations that are either inherited from affected parent or formed in the parental gamete prior to conception affect all cell populations (“germline” mutations). In such cases, cells with an additional *PTEN* mutation (a “second hit”) results in hamartomata and cancer predisposition. The two-hit theory, also referred to as Knudson hypothesis, also explain the nature behind the observed segmental overgrowth: affected individuals are *susceptible* to Akt overactivation with only one active allele of *PTEN*; a second spontaneous deactivating mutation in *PTEN* in a post-zygotic developing tissue may occur, leading to discretely affected areas. The segmental overgrowth is manifested differently in each phenotype of PHTS: newborns with Bannayan–Riley–Ruvalcaba have striking macrocephaly (≥4.5 SD), out of proportion to their birth weight and length; those with Proteus-like presentation exhibit mosaic pattern of rapidly progressive overgrowth of different tissue types; Cowden syndrome, typically manifests in the second decade of life, and is associated with hamartomata and macrocephaly. Cowden syndrome bears an increased lifetime risk of benign and malignant tumors in the breast (85% malignancy risk), papillary or follicular thyroid carcinoma (35%), renal cell carcinoma (35%), endometrial (28%), colorectal (9%), melanoma (5%), and rarely Lhermitte–Duclos disease (cerebellar dysplastic gangliocytoma).

Bannayan–Riley–Ruvalcaba is further characterized by hypotonia, intellectual disability (50–70%), proximal myopathy (60%), scoliosis (50%), hamartomatous polys in colon (45%), which may cause intussusception or rectal bleeding, seizures (25%), and joint hypermobility. A distinctive clinical finding in most affected individuals is pigmented macules on the penile shaft and if seen on physical examination should raise immediate concern for PHTS. The risk for tumors and malignancy is currently perceived to be similar to Cowden syndrome, especially breast and thyroid cancers, and merits similar surveillance. Proteus-like syndrome is clinically similar to Proteus syndrome and exhibit vascular malformations, lipomas, connective tissue nevi, epidermal nevi, and cranial hyperostosis. Cowden syndrome, named after the first reported patient, is rarely expressed in children and has distinctive trichilemmomas (benign neoplasm derived from the outer root sheath epithelium of the hair follicle), papillomatous papules (benign neoplasm of epithelium), and acral and plantar keratosis seen in 99% of patients by the third decade of life.

Mutations in *PTEN* (10q23), can give rise to either one of the above phenotypes—making these three conditions allelic disorders. Furthermore, it has been shown that the very same *PTEN* variant in an affected family can be expressed as either Cowden or Bannayan–Riley–Ruvalcaba syndrome in different family members. Another allelic disorder is macrocephaly with autism. In fact, 10–20% of autistic children with macrocephaly harbor germline mutation in *PTEN* *(**89**)*. Figure 3 shows a patient with PHTS who presented with a “hard-to-miss” congenital macrocephaly. Macrocephaly is seen with other overgrowth syndrome, including Sotos, Weaver, and familial cases, however, when presented with either benign cutaneous growth (Cowden syndrome), very severe macrocephaly, penile macules (Bannayan-Riley-Ruvalcaba), or with autism, it is suggestive of PHTS and warrants a referral to geneticist for further evaluation. We encourage clinicians to routinely measure fronto-occipital circumference (FOC) during well-child checks, as macrocephaly can often be missed by inspection alone, especially among overweight patients when head circumference is perceived as “proportional” to the body habitus.

Genetic testing for *PTEN* includes gene sequencing, analysis of deletions and duplications and the promoter region. PHTS is an autosomal dominant disorder, meaning that only one pathogenic variant in *PTEN* gene is enough to express the disorder. Affected individuals have a 50% chance of transmitting the pathogenic variant to each one of their children. We recommend referring to genetics post-pubertal individuals for discussion about family planning. *In vitro* fertilization (IVF) techniques with preimplantation genetic testing for monogenic disorder (PGT-M) for fetuses can be offered to significantly reduce the transmission risk.

Management of PHTS include symptomatic management for the mucocutaneous manifestations of CS (which resembles management of warts–5-fluorouracil, curettage, cryosurgery or laser surgery) and close follow up on development with neuropsychological evaluation (including IQ test) if suspicion for intellectual disability arises. Genetic testing for other family members is also recommended (33, 89–91). For confirmed Cowden or Bannayan–Riley–Ruvalcaba syndromes (i.e., affected individuals that do not express the Proteus-like segmental overgrowth or are presented with macrocephaly and autism), specific cancer surveillance guidelines have been published:

For women, increased risk for breast cancer merits similar management to *BRCA1* or *BRACA2* carriers: clinical breast examination starting at 25 years of age or 5–10 years before the earliest known breast cancer in the family (whichever comes first). Starting at age 30–35 or 5–10 years prior to first known breast cancer in the family, annual mammography, tomosynthesis (“3D mammography”), or MRI with contrast should be performed until the age of 75. A discussion about risk reduction with double mastectomy should be conducted (sparing oophorectomy). Endometrial cancer screening is symptom-based (menstrual cycle irregularities, post-menopausal bleeding) only. For both men and women, colonoscopy should be performed at least every 5 years starting at 35 years of age or 5–10 years prior to first known familial case (whichever comes first). Neck ultrasound for thyroid cancer should be obtained at the age of 7, then if negative every 2 years. Renal US is recommended at 40, then every 1–2 years subsequently (92, 93).

### PIK3CA-Related Segmental Overgrowth

**Figure 4:**
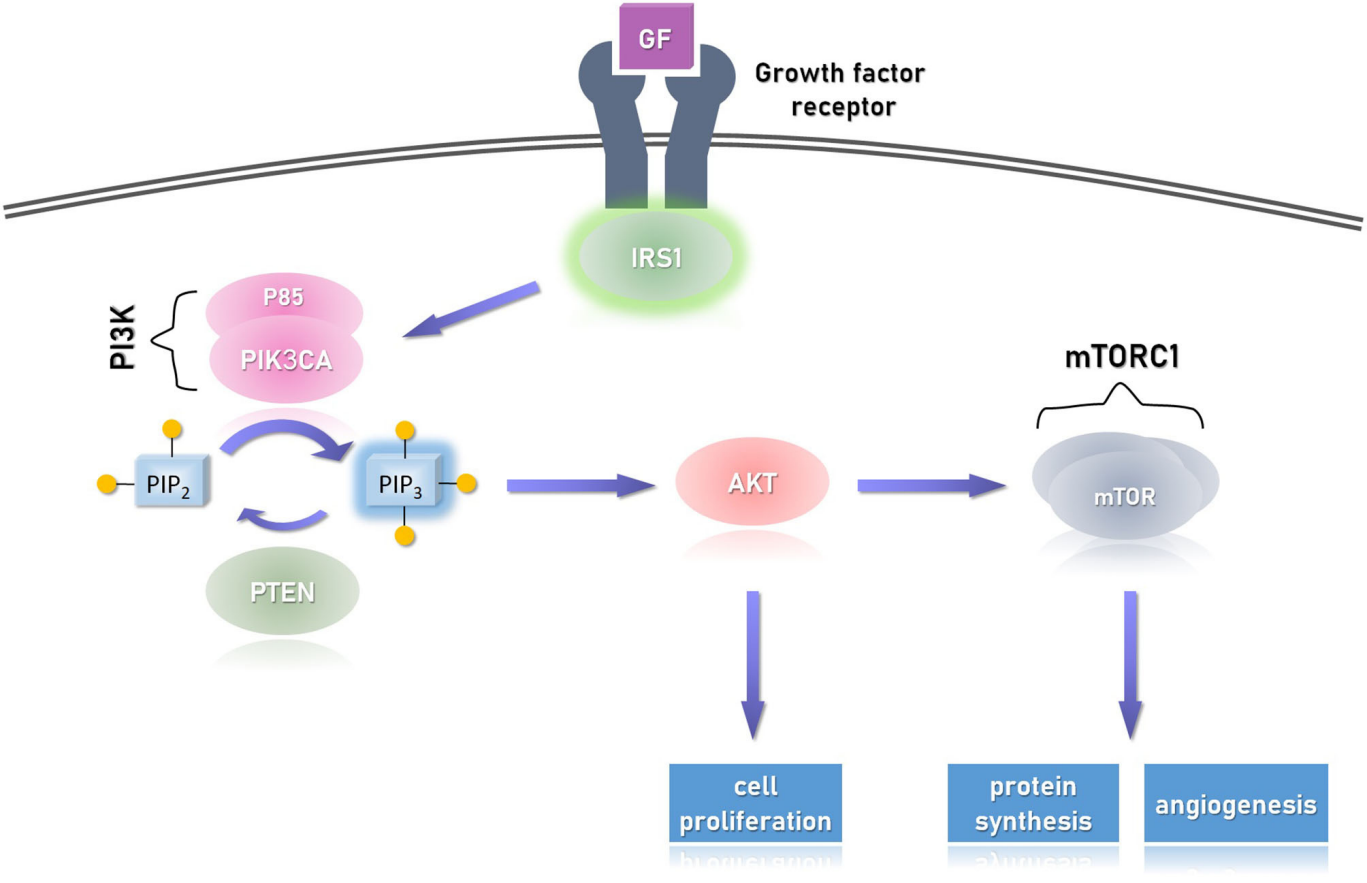
The cellular response to growth factor (GF) via its receptor. Upon dimerization of the receptor, IRS1 (insulin receptor substrate 1) is phosphorylated and activates (via its SH2 domain) downstream effectors, particularly PI3K (phosphatidylinositol 3-kinase). The latter, in turn, phosphorylates the second messenger PIP2 (phosphatidylinositol 4,5-bisphosphate), resulting in the activation of AKT (protein kinase B), which activates the mTORC1 (mammalian target of rapamycin complex 1). This pathway promotes cellular proliferation (via AKT) and also promotes angiogenesis and protein synthesis via the mTORC1 effector. Overactivation of the catalytic unit of PI3K, called PIK3CA, or AKT1 may result in uncontrolled activation of this pathway and signal-independent (over) growth. The former is seen in PIK3CA-related overgrowth spectrum (PROS) and the latter in Proteus syndrome, both are segmental overgrowth syndromes. A similar picture can be seen with biallelic deactivation of PTEN which is a growth repressor, as it dephosphorylates PIP_3_ back to its inactive form PIP_2_. This condition is seen in PTEN hamartoma tumor syndrome (PHTS). Not shown in the figure, but similar to PHTS, other growth repressors are the TSC1/2 complexes (tuber sclerosis complex), which inhibit mTORC1, but themselves are inhibited by AKT. The pathogenesis of variants in TSC1/2 is different, resulting in discrete tuberous growth of the cutaneous and CNS tissues, and predispose to variety of cancers. Of note, the PI3K/AKT/mTOR pathway is one pathway in which the growth factor activates.

As depicted above, germline mutations in *PTEN*, leading to only one functional copy of this gene, create susceptibility for the complete loss of PTEN function by a second spontaneous mutation in any tissue. Such an event creates a pattern of affected tissues interspersed between healthy tissues, referred to as mosaicism. When testing the DNA of individuals with PHTS, leukocytes will demonstrate one variant (one mutated allele) of *PTEN*, while testing cells from affected tissue will show variants in the two alleles. On the contrary, proteins that participate in the growth-promoting pathway that PTEN inhibits, may harbor only one variant which allows their escape from inactivation, resulting in overgrowth. PTEN is the opposed enzyme of phosphatidylinositol 3-kinase: the former removes and the latter adds phosphate to the aliphatic second messenger phosphatidylinositol di/tri-phosphate. The catalytic subunit of phosphatidylinositol 3-kinase is p110α, encoded by the gene *PIK3CA*. Variants causing overactivation of p110α shifts the balance between p110α and PTEN toward creation of phosphatidylinositol (3,4,5)-trisphosphate, which activates AKT (see Figure 4). Unlike *PTEN*, mutations in *PIK3CA* are sporadically occurring: they can occur early in embryo and will thus cause all tissues differentiated from that stem cell to over-proliferate causing a segment of overgrowth; or they can occur in a mature tissue, which is associated with malignancy. When examining the blood or saliva from these patients, generally no mutation will be found; mutation can be detected only when sampling the affected tissues. It is thought that germline mutations in these proteins will cause severe generalized overproliferation that is not compatible with life: mice with diffuse p110α over-activation rapidly die of intraabdominal hemorrhage (94). The phenotype of PROS is of a segmental, disfiguring, asymmetric tissue overgrowth; its severity depends on the level of mosaicism—the balance between the affected and unaffected cells. Two *PIK3CA* syndromes are recognized. The first is CLOVES (Congenital Lipomatous Overgrowth, Vascular malformations, Epidermal naevi, Scoliosis/skeletal and spinal syndrome) and the second is megalencephaly-capillary malformation (MCAP) syndrome. CLOVES is associated with complex lipomatous overgrowth of the thoracic and abdominal wall, macrodactyly (enlargement of a digit), and plantar or palmar overgrowth which results in wrinkling of the overlying palmar or plantar skin. The lipomatous masses can be found on the skin or extending into the paraspinal and intraspinal spaces which may cause compression of the cord or nerve roots. The lesions are commonly covered by capillary, venous, lymphatic, or arteriovenous malformations. Skeletal malformations can be severely deforming; it includes scoliosis and asymmetric bony overgrowth. Structural anomalies are found in the CNS (and are associated with variable degree of intellectual disability) and kidneys. A very similar presentation of asymmetric overgrowth of bony, adipose, fibrous, and vascular overgrowth due to PIK3CA over-activation is called Fibroadipose hyperplasia. Milder presentation may include only asymmetric overgrowth of limbs with overlying vascular malformations and can be expressed merely as isolated macrodactyly (95).

MCAP syndrome is comprised of megalencephaly or hemi-megalencephaly (increased parenchymal volume of the entire or one hemisphere of the brain) seen perinatally by elevated occipitofrontal circumference. It is accompanied by secondary overgrowth of specific brain structures, and ventriculomegaly with polymicrogyria (excessive small gyri) are common. Patients with MCAP are hypotonic and prone to seizures (30%). Similarly to CLOVES, vascular malformations are common (although more confined to the skin) and asymmetric overgrowth of limbs may be seen. Not surprisingly, mutations causing CLOVES and MCAP syndromes are seen in the COSMIC database, as *PIK3CA* is commonly overactivated in many common cancers. However, it is not clear that PROS has an increased risk for tumors and no specific surveillance guidelines are established yet (49). Management of these patients is symptom-based—debulking surgeries for lipomata, orthopedic referral for scoliosis, and standard treatment for seizures; CNS imaging for brain abnormalities and paraspinal lipomatous growth; and close follow-up on development (95). Experimental inhibitors for either PIK3CA, or downstream effectors AKT or mTORC1 are being investigated with promising results (94, 96). Sirolimus, an allosteric mTORC1 inhibitor has shown reduction of up to 7% in the volume of affected tissue without effect on unaffected tissues; however, more than a third of the patients can develop serious side effects which may limit its use (97). In two recent publications involving three cases, Alpelisib, a p110α inhibitor, was tried after the failure of sirolimus and was found to show significant improvement in all subjects, including shrinkage of tumors, reduction of capillary malformations and epidermal nevi, and even cognitive improvement (94, 98). Several other PI3K/AKT/mTOR pathway inhibitors are in different stages of clinical trials, summarized by Hillmann and Fabbro (99). A dual PIK3A/mTOR inhibitor, Dactolisib, is currently under investigation, showing reduction in endothelial proliferation *in vitro* (100).

Genetic testing should be obtained from the affected tissue and not blood. Prognosis depends on the degree of the severity of the phenotype ranging from guarded to dismal (101). Rarely the somatic mutation in *PIK3CA* is expressed in gonads; therefore, transmission to next generation is expected to be unlikely. Similarly, because this is a post-zygotic mutation, the risk for recurrence in a family is not elevated.

### Other Segmental Overgrowth Syndromes Associated With Vascular Malformation

Somatic overgrowth with overlying vascular malformation is shared by many other syndromes making this clinical finding an important sign for an underlying disorder. Combined capillary, venous and lymphatic malformation (all considered slow-flow malformations) along with limb enlargement is seen in Klippel–Trenaunay syndrome (KTS); arteriovenous (fast-flowing) fistulae without lymphatic malformation along an enlarged limb are seen in Parkes–Weber syndrome (facial capillary malformation with occasional mild hypertrophy of the maxilla is seen in the closely related Sturge–Weber syndrome). In KTS, the affected limb is the lower extremity in 95% of cases and upper extremity in close to 5%. It is uncommon to have hypertrophy of the trunk. Capillary malformations appear bluish-purplish in color and may extend to the trunk but rarely to the face. They are commonly accompanied by lymphatic malformation causing lymphatic leak and lymphedema of the involved extremity. In 80% of patients, significant varicosities extending from the dorsum of foot to the popliteal or even the gluteal venous system are noticeable beginning in infancy or early childhood. It is often complicated by thrombophlebitis (up to 50%) and pulmonary embolism (10%). Surgical correction may be complicated by incompetency of the deep vein system seen in some patients. The affected limb may exhibit bone and/or soft tissues hypertrophy and thus the limb can be asymmetric in both length and girth (the latter also affected by the lymphatic malformation). Toes maybe significantly more affected than the rest of the limb. The sole may have wrinkled appearance to it but lacks the firm consistency of cerebriform connective tissue nevi seen in Proteus syndrome (33, 102). Closely resembling is Parkes-Weber syndrome (PWS) in which vascular malformations and tissue hypertrophy is seen in the lower extremity in about 90% and upper extremity in about 10%, however arteriovenous (AV) fistula are persistently presented and lymphatic malformations are absent. Due to the AV fistula, high-output heart failure can develop in about 31% of cases and almost 10% manifest distal arterial ischemia, making the overall prognosis of this syndrome worse than KTS (103). Despite similar presentation, the genetic basis of these two syndromes differs. In KTS, mutation in one copy of PIK3CA was recently reported to occur in 19 out of 21 cases (104); however, it is yet to be established if KTS is truly a part of PROS described above. In PWS, the genetic alteration involves the RASA1 gene encoding Ras p21 protein activator 1, involved in capillary malformation–arteriovenous malformation (CM-AVM) syndrome (105). Similar to PROS, these syndromes are sporadically occurring and are not familial.

## Conclusions

Overgrowth syndromes can present with excessive post-natal growth, tall stature that is out of proportion to the individual's genetic potential in a child, isolated increased head circumference, or somatic asymmetry. Clues for overgrowth syndrome can come from the following: (i) physical examination such as omphalocele, abnormal ear creasing, and macroglossia for BWSp, dolichocephaly and frontal bossing in Sotos syndrome or lipomas, cerebriform cutaneous nevi or vascular anomalies in segmented overgrowth syndrome; (ii) imaging findings such as accelerated bone age and visceromegaly in the prenatally presenting overgrowth syndromes, or hemi-megalencephaly and paraspinous lipomas seen in PIK3CA-related overgrowth; and (iii) neurobehavioral assessment showing learning difficulties, developmental delays, or autism as seen commonly in Sotos, Weaver, and PTEN-hamartoma syndromes. Overgrowth syndromes can predispose affected individuals to hypoglycemia, embryonal tumors, seizures, developmental delay, intellectual disability, and musculoskeletal complications; therefore, timely diagnosis is important. Careful history taking, physical examination, anthropometric measurements, and developmental follow-ups, all of which are tools used on routine office visits, can provide significant evidence for suspected overgrowth syndrome. Maintaining a high index of suspicion for these disorders can assist in timely referrals to genetics, which can assist in the evaluation and testing of these patients. We encourage the primary clinician to be alert of the “large” end of the weight, length and FOC measures, and become familiar with BWSp, which is the most common overgrowth syndrome requiring vigilant tumor surveillance (see Table 1).

### Summary

- Based on expert opinion, stature larger than 2 standard deviations from the mean should be considered tall and raise suspicion for an overgrowth syndrome.
- Based on expert opinion, workup for tall stature that exceeds the individual's mid-parental height should include assessment of growth velocity and should consider full blood counts, complete biochemical analysis, IGF-I, IGFBP-3, free T4, and TSH, as well as a karyotype and bone age.
- Based on expert opinion, newborns with either macroglossia, exophthalmos, lateralized overgrowth, persistent hyperinsulinism, multifocal or bilateral Wilms tumor, or adrenal cortex cytomegaly should be tested molecularly for Beckwith–Wiedemann syndrome.
- Based on published guidelines, individuals with BWSp or SGB syndrome should undergo routine cancer surveillance every 3 months for hepatoblastoma (until the age of 4) and Wilms tumor (until the age of 7).
- Based on expert opinion, individuals with FOC > 4 SD and autism should be tested molecularly for possible PHTS.
- Based on published guidelines individuals with PTEN-hamartoma Tumor syndrome should undergo breast cancer surveillance similar to BRCA 1/2, colonoscopy surveillance every 5 years, biennial thyroid US, renal US every 1–2 years.
- Based on expert opinion, individuals with intellectual disability and tall stature should be evaluated for suspected overgrowth syndrome.

## Author Contributions

JM conceptualized, prepared, and wrote the manuscript and made the tables and figures. SL reviewed, edited, and revised the manuscript and provided the patients' figures.

## Conflict of Interest

The authors declare that the research was conducted in the absence of any commercial or financial relationships that could be construed as a potential conflict of interest.
