# Corrigendum: Overgrowth Syndromes—Evaluation, Diagnosis, and Management

**DOI:** 10.3389/fped.2020.624141

**Published:** 2020-12-23

**Authors:** Joshua Manor, Seema R. Lalani

**Affiliations:** Department of Molecular Genetics, Baylor College of Medicine, Houston, TX, United States

**Keywords:** overgrowth, Beckwith-Wiedemann, Simpson-Golabi-Behmel, Sotos, Weaver, Pten, PIK3CA, Proteus Syndrome

In the original article, there was a mistake in **Figure 3** as published. The permission for use of copyrighted material is not yet available to the authors. This figure has therefore been removed and referenced appropriately. Due to the removal of Figure 3, the figure numbering has been updated.

Additionally, in the original article, there was a mistake in Table 1 as published. The term “Exophthalmos” is incorrect, and should be replaced by the correct term, “Exomphalos.” The corrected Table 1 appears below.

**Table 1:** Clinical diagnostic criteria for Beckwith–Wiedemann syndrome.

**Feature**	**Pts**
**Cardinal findings**	
Macroglossia	2
Exomphalos	2
Lateralized overgrowth	2
Multifocal and/or bilateral Wilms tumor	2
Persistent hyperinsulinism (> 1 week)	2
Characteristic pathology: adrenal cortex cytomegaly, placental mesenchymal dysplasia, pancreatic adenomatosis	2
**Minor findings**	
Birthweight > 2 SD above the mean	1
Facial naevus simplex	1
Polyhydramnios	1
Ear creases and/or pits	1
Transient hyperinsulinism (<1 week)	1
Characteristic tumor: Unilateral Wilms tumor, neuroblastoma, rhabdomyosarcoma, hepatoblastoma, adrenocortical carcinoma, or pheochromocytoma	1
Nephromegaly and/or hepatomegaly	1
Umbilical hernia and/or diastasis recti	1
**Interpretation:**	
**Clinical score**	**Diagnosis**
4+	BWSp confirmed
2–3	Diagnosis by genetic testing
0–1	BWSp rejected

*Adapted from Brioude et al. 2018*.

There was one final error in the original article. “SET2D-related disorder” is incorrect, and this should be written as “SETD2-related disorder”. A correction has been made to Overgrowth Syndromes Presenting Prenatally, Sotos Syndrome, Paragraph 6: SETD2-related disorder is another example of a Sotos-like autosomal dominant overgrowth syndrome resulting in post-natal overgrowth, macrocephaly, prominent forehead, and advanced bone age, named Luscan-Lumish syndrome (OMIM 616831), (70), adding another layer of complexity to a diagnosis of Sotos syndrome based on clinical symptoms alone.

The authors apologize for these errors and state that this does not change the scientific conclusions of the article in any way. The original article has been updated.

